# Opportunities Offered by Graphene Nanoparticles for MicroRNAs Delivery for Amyotrophic Lateral Sclerosis Treatment

**DOI:** 10.3390/ma15010126

**Published:** 2021-12-24

**Authors:** Benedetta Niccolini, Valentina Palmieri, Marco De Spirito, Massimiliano Papi

**Affiliations:** 1 Dipartimento di Neuroscienze, Università Cattolica del Sacro Cuore, 00168 Rome, Italy; benedetta.niccolini01@icatt.it (B.N.); marco.despirito@unicatt.it (M.D.S.); massimiliano.papi@unicatt.it (M.P.); 2 Fondazione Policlinico Universitario “A. Gemelli” IRCSS, 00168 Rome, Italy; 3 Istituto dei Sistemi Complessi, CNR, Via dei Taurini 19, 00185 Rome, Italy

**Keywords:** amyotrophic lateral sclerosis, miRNAs, therapy, nanoparticle, delivery systems, graphene

## Abstract

Amyotrophic lateral sclerosis (ALS) is characterized by the degeneration and death of motor neurons. This neurodegenerative disease leads to muscle atrophy, paralysis, and death due to respiratory failure. MicroRNAs (miRNAs) are small non-coding ribonucleic acids (RNAs) with a length of 19 to 25 nucleotides, participating in the regulation of gene expression. Different studies have demonstrated that miRNAs deregulation is critical for the onset of a considerable number of neurodegenerative diseases, including ALS. Some studies have underlined how miRNAs are deregulated in ALS patients and for this reason, design therapies are used to correct the aberrant expression of miRNAs. With this rationale, delivery systems can be designed to target specific miRNAs. Specifically, these systems can be derived from viral vectors (viral systems) or synthetic or natural materials, including exosomes, lipids, and polymers. Between many materials used for non-viral vectors production, the two-dimensional graphene and its derivatives represent a good alternative for efficiently delivering nucleic acids. The large surface-to-volume ratio and ability to penetrate cell membranes are among the advantages of graphene. This review focuses on the specific pathogenesis of miRNAs in ALS and on graphene delivery systems designed for gene delivery to create a primer for future studies in the field.

## 1. Introduction

**Figure 1:**
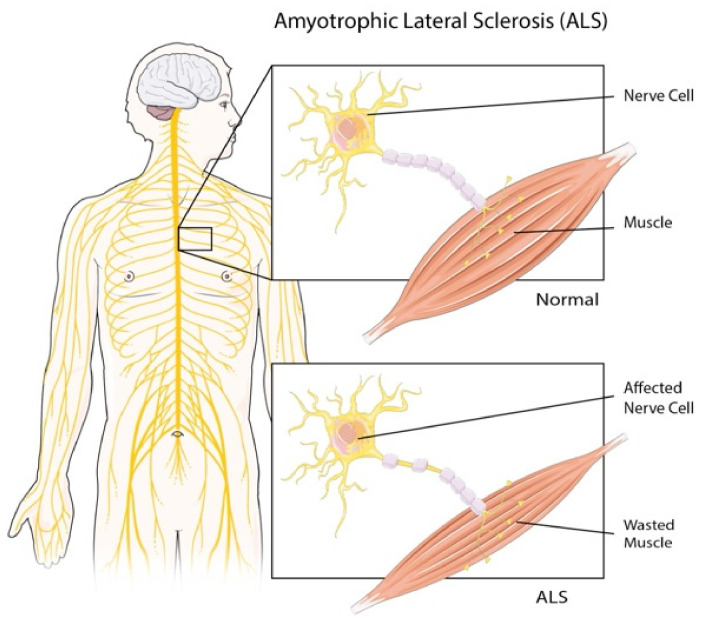
Amyotrophic lateral sclerosis. The motor neurons degeneration in ALS causes muscular atrophy [3].

Amyotrophic lateral sclerosis (ALS), known also as motor neuron disease or Lou Gehrig’s disease, is a neurodegenerative affection caused by the degeneration and the death of motor neurons, responsible for carrying out muscle movements [1,2]. The progressive motor neurons degeneration causes muscle atrophy, paralysis, and respiratory failure [3] (Figure 1).

According to recent studies, the rate of ALS is about 1 to 3 patients per 100,000 worldwide [3] with an average life span from diagnosis of 24 to 48 months [3]. Historically, ALS forms can be categorized into two groups: the familial form of ALS (fALS), and sporadic ALS form (sALS) [4]. fALS represents 5–20% of all ALS cases while the remaining portion of the cases are considered sALS forms [2]. Even if the mechanisms of neuronal death are still unclear, it is hypothesized that ALS is caused by an intricate relation involving cellular, molecular, and genetic pathways. Some probable causative factors have been proposed for contributing to ALS onset.

**Figure 2:**
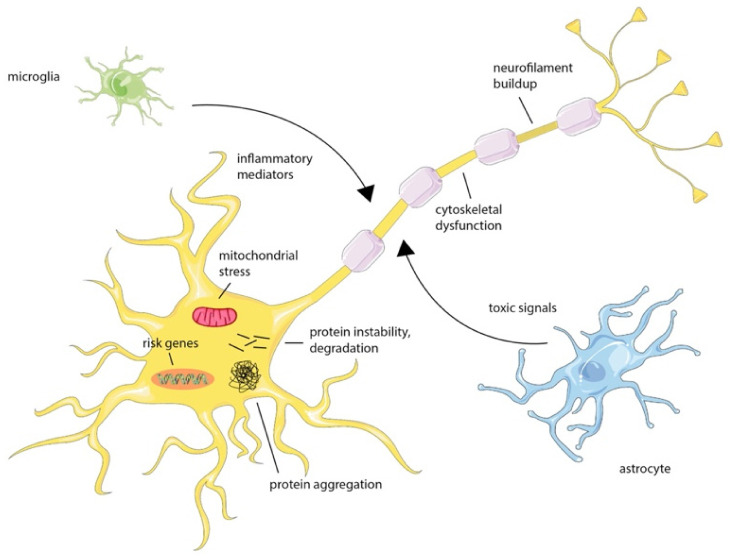
Cellular alterations of ALS. Molecular causes of ALS are still unknown, even if different cell types in the brain, such as microglia and astrocytes, are thought to contribute to neuronal dysfunction. The protein aggregation or degradation alters several pathways in the neurons.

First of all, mutations in genes cause an alteration of the normal protein function. Over 20 genes have been related to ALS, among these, mutations on S*od1*, *tardbp*, *fus*, *c9orf72*, and *pfn1* cause ~60–70% of the fALS cases and 10% of the sALS cases. The GGGGCC hexanucleotide expansion mutation in the 5′ noncoding region of *c9orf72* nuclear protein is undoubtedly the most common genetic alteration of ALS [5,6]. Protein aggregation, glutamate excitotoxicity, endoplasmic reticulum and oxidative stress, neuroinflammation, mitochondrial dysfunction, loss of trophic factors, and defects in axonal transport are proposed mechanisms that promote ALS progression [1] (Figure 2). The *c9orf72* mutation causes both gain-of-function and loss-of-function mechanisms that alter neuronal, glial, and immune cell function. Because loss-of-function mechanisms impair autophagy and gain-of-function mechanisms produce aggregation-prone proteins, this genetic defect induces both of the predominant neurotoxic mechanisms causing ALS.

There is no resolutive pharmacological treatment for ALS, even if the glutamatergic neurotransmission inhibitor Riluzole and the antioxidant drug Edaravone are found to be effective in early ALS stages and are the only drugs approved for the treatment of patients with ALS [7].

In recent years, the search for new therapeutic targets for the treatment of ALS has emphasized the role of some nucleic acids known as microRNAs (miRNAs), specific non-coding RNAs (ncRNAs) that vary in many biological fluids such as blood and/or cerebrospinal fluid in ALS patients [8]. Some miRNAs play an important role in the development and in the function of the motor neurons. Alterations of these functions contribute to ALS neurodegeneration [9] and disease progression [10]. Since there is a differential expression of specific miRNAs during ALS pathogenesis, these small ncRNAs are studied as diagnostic biomarkers [11]. Moreover, comforting results in animal models have proved the role of miRNAs as therapeutic agents. For this reason, new therapies and miRNAs delivery systems are being designed for ALS. This review is focused on the analysis of the main mechanisms of miRNA biogenesis and malfunctioning in ALS and on the range of possible delivery strategies offered by nanoparticles for miRNA targeting. Additionally, the possibility of designing delivery strategies with graphene, a bidimensional nanomaterial with a high surface area and unique biological effects, will be discussed.

## 2. MicroRNAs: Biogenesis, Function, and Mechanism of Action

Three decades ago, the scientific community was centered on the study of the coding deoxyribonucleic acid (DNA) however, after genome transcription, it became clear that due to the wide range of ncRNAs, a novel concept of gene regulation involving regulatory RNAs including miRNAs had to be established.

MiRNA is a group of small ncRNAs [12,13] with an average length of 19 to 25 nucleotides, involved in the regulation of gene expression. These ncRNAs act by eliciting translation repression or promoting messenger RNA (mRNA) decay [14,15]. By binding the 3′ untranslated regions (UTR), the coding sequences, or the 5′ UTR, miRNAs inhibit mRNA translation or promote its degradation [16,17].

MiRNAs are fundamental in a variety of biological processes [18] since most genes are regulated by miRNAs at both post-transcriptional and translational levels [14]. To understand the potential of miRNAs, it is necessary to describe their biogenesis.

In most cases, the biogenesis of miRNAs is a multi-step process that starts in the nucleus, where these small ncRNAs are transcribed as unstructured precursors, termed primary miRNAs (pri-miRNAs) [19] by RNA polymerase II.

**Figure 3:**
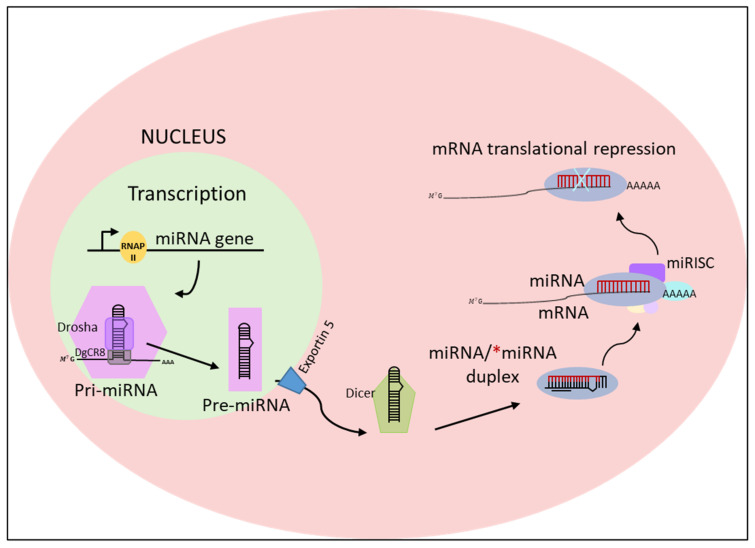
A schematic representation of miRNA biogenesis and function in eukaryotic cells. The biogenesis of a miRNA starts in the nucleus by RNA polymerase II, from a large 7-methylguanosine (m7G) capped and polyadenylated (poly(A) tail) transcript (pri-miRNA). This precursor is then processed by the RNase III enzyme Drosha and its cofactor Dgcr8 into a stem-looped structure called pre-miRNA. The pre-miRNA is then exported by exportin 5 into the cytoplasm where it becomes a mature miRNA after being processed by another Rnase III called Dicer. This process requires the removal of the final loop, resulting in a mature miRNA duplex. The mature miRNA binds the miRNA-induced silencing complex (miRISC), where base-pairing between the seed-sequence of miRNA and complementary sequences of mRNAs results in post-transcriptional gene silencing.

Pri-miRNAs have a length of several kilobases and contain an RNA hairpin in which one of the two strands includes the mature miRNA [20]. Pri-miRNAs often have well-conserved terminal loops where auxiliary factors bind to ensure processing. One of these factors is the heterogeneous nuclear ribonucleoprotein, which binds the loop of the pri-miRNA to produce a relaxation at the stem [21,22]. At this point, the biogenesis occurs with the excision of the upper part of the RNA hairpin by the nuclear RNase III enzyme Drosha, which acts in concert with the DiGeorge Syndrome Critical Region 8 (DIGCR8) complex to generate 60–80 base pair chains with typical hairpin conformation know as precursor miRNAs (pre-miRNA) (Figure 3) [19,23,24,25]. Pre-miRNAs are sequentially processed in the cytoplasm, to mature miRNAs. First, the nuclear export factor Exportin 5 allows the export of the pre-miRNA to the cytoplasm [26,27,28,29]. In the cytosol, the RNase III enzyme Dicer cuts the terminal loop of the pre-miRNA to produce a ~20-bp RNA duplex with 2-nt 3′ overhangs [19,30,31,32]. These duplex RNAs bind the protein Argonaute, which separates the double chain and is then incorporated into a large protein complex, the RNA induced silencing complex RISC, towards complementary mRNA targets [33,34,35]. Only the final miRNA can silence specific genes by targeting the corresponding mRNA.

Analysis of the genomic distribution miRNAs has demonstrated that the majority of them is located between genes, in intergenic regions, and sometimes in clusters [24,36,37]. Clusters are transcribed together from physically adjacent miRNAs and show similar expression profiles [38,39]. miRNAs broadly impact homeostasis and their abnormal expression can be related to various diseases [40,41,42,43]. In the following section, the role of miRNAs analysis in ALS diagnosis will be discussed.

## 3. MiRNAs Dysfunction in ALS and Their Uses as Biomarkers of Disease

Several miRNAs have been identified to be crucial for ALS development through different pathologic mechanisms. MiRNA alterations in animal models and patients are resumed in Table 1.

The dysfunctions in microRNAs in ALS cause, among others, increased inflammation [44], selective suppression of neurofilament light chain (NFL) mRNA [49], endoplasmic reticulum dysfunctions [58], oxidative stress, disruption of axonal transport, and cytoskeleton and mitochondrial defects [59]. MiRNAs can be considered robust biomarkers for the early diagnosis of ALS, since, in contrast to other biomarkers, miRNAs can be simply secreted into the extracellular space in the central nervous system (CNS). A high amount of information about ALS has been derived from Superoxide Dismutase 1 (SOD1)-linked cellular models and SOD1-G93A transgenic mouse, which can simulate the pathological phenotype [60].

In the SOD1-G93A mouse, the upregulation of miRNA-365 and miRNA-125b have the effect of increasing tumor necrosis factor α (TNFα) by acting on interleukin (IL)-6-signal transducer and activator of transcription 3 (STAT3) pathway and TNFα synthesis. MiRNA-365 and miRNA-125b, found overexpressed in SOD1-G93A mouse microglia, work on the repression of the IL-6-STAT3 pathway and promote the activation of TNFα gene transcription. In addition, the TNFα released in the medium acts by upregulating miRNA-125b. These phenomena cause the switching of microglia toward a detrimental phenotype and cause global neuroinflammation [44,61]. In the same transgenic model, the downregulation of miRNA-124 plays a crucial role in astrocytic differentiation, since it reduces the translation inhibition of Sox2 and Sox9 factors. Both these transcription factors have crucial roles in astrocyte development: the overexpression of Sox2 suppresses neuronal progenitor cells differentiation. Sox9 also plays an essential role in the maintenance of multipotent neural stem cells [45]. In the CNS, Sox9 blocks differentiation into neurons and supports glial development in the peripheral nervous system [62]. The downregulation of miRNA-124 (which targets Sox9) has been shown to increase Sox9 expression and decrease neurogenesis [63]. Additional studies have reported that in the SOD1-G93A mouse, the miRNA-218 is enriched in the motor neurons and can be released in the extracellular space [46]. This release downregulates the expression of the excitatory amino acid transporter 2 (EAAT2), one of the major glutamate transporters expressed predominantly in astroglial cells [64]. The deregulation of EAAT2 may lead to increased synaptic glutamate, which causes excessive glutamate signaling and death of post-synaptic neurons [65].

Another miRNA deregulated in the SOD1G93A mouse model is miRNA-193b-3p. Its downregulation stimulates cell death by targeting tuberous sclerosis 1 [47], which controls rapamycin complex 1, a regulator of autophagy, and a neuroprotector [66,67]. Finally, in recent research conducted on the SOD1G93A mouse model, miRNA-206 was identified as a potential circulating biomarker of the disorder as it exhibited strong upregulation in the serum during the pre-symptomatic stages [68,69].

Marlena and colleagues have seen that in the sALS wobbler mouse, a model which shows almost all the clinical features of human ALS patients, miRNA-375-3p downregulation produces an inefficient p53 inhibition, increasing ROS production, and inducing cell death [48].

In patients, miRNAs can be clinically detected and then quantified from many biological fluids, such as cerebrospinal fluid, serum/plasma, saliva, and urine [70]. Campos-Melo et al. found unique miRNAs expression profiles in spinal cord lysate of sALS patients (miRNA-146a*, miRNA-524-5p, and miRNA-582-3p). These dysregulated miRNAs control the NFL mRNA 3′UTR, with critical repression and aggregation in the ALS spinal motor [49]. Another study focused on the control of neuronal function and the synaptic formation by astrocytes [51], where extracellular vesicles genesis and miRNA load are dysregulated in C9orf72 mutated human astrocytes. In particular, the downregulation of miRNA-494-3p negatively regulates semaphorin 3A and other targets related to axonal maintenance.

Moreover, mutation of the RNA-binding protein FUS, which leads to motoneuron loss and is associated with decreased miRNA-375 levels in humans. miRNA-375 has the role of targeting the ELAVL4 factor, an RNA-binding protein and apoptotic factor, aberrantly increased in FUS mutant motoneurons [50]. Chen et al. carried out an analysis in leukocytes obtained from patients with sALS, studying the expression profile of 1733 human miRNAs. They discovered deregulation of miRNA-183, miRNA-193b, miRNA-451, and miRNA-3935 relative to healthy controls71. The study of De Felice and colleagues highlighted the different expressions in the neuromuscular junction of miRNA-223-3p, miRNA-338-39, and miRNA-326, which were all up-regulated in the sALS patients compared to the healthy controls [52].

Another study investigated the correlations between serum miRNAs and some ALS clinical parameters, in both sALS and fALS. Seven miRNAs have been found upregulated (miRNA-1, miRNA-19a-3p, miRNA-133a-3p, miRNA-133b, miRNA-144-5p, miRNA192-3p, and miRNA-192-5p) and six downregulated (let-7d3p, miRNA-139-5p, miRNA-320a, miRNA-320c, miRNA320b, and miRNA-425-5p) [53]. Anika et al. have identified the downregulation of microRNA-1825 in CNS and extra-CNS system organs of both sALS and fALS populations [54]. A minor level of microRNA-1825 led to the over translational of tubulin-folding cofactor b (TBCB) which causes the depolymerization and the degradation of tubulin alpha-4A (TUBA4A). The upregulation of TBCB and the downregulation of TUBA4A protein was underlined in brain cortex tissue of fALS and sALS patients and provoked the motor axon defects. The finding of the miRNA-1825/TBCB/TUBA4A pathway exposes a pathogenic mechanism in both fALS and sALS [54].

Other interesting studies aimed to identify skeletal muscle miRNAs (myo-miRNA) as potential biomarkers for ALS in patients. One study revealed that miRNA-424 and miRNA-206 were significantly overexpressed both in the skeletal muscle and in the plasma of ALS patients, and it has been suggested that the baseline serum levels of the two miRNAs are related to slow changes in medical research council sun scores in ALS patients [55]. Myo-miRNAs are related to muscle proliferation, differentiation, shaping, and repair. Pegoraro and colleagues tested the level of five myo-miRNAs (miRNA-1, miRNA-27a, miRNA-133a, miRNA-133b, and miRNA-206) in ALS patients. They found that the levels of four of them (miRNA-1, miRNA-27a, miRNA-133a, miRNA-206) were higher in those patients characterized by an early disease onset of ALS. Moreover, lower myo-miRNAs levels have been related to a shorter disease period in ALS patients, and represent a novel prognostic indicator of disease [41].

Furthermore, some myo-miRNAs levels are significantly lower after physical training. Patients with ALS often avoid exercise to take care of muscle strength and to reduce possible muscle overload, but animal models and human studies underline the benefits of programmed workouts to delay disease progression and symptoms [71,72]. Decreased miRNA-1, miRNA-133a, miRNA-133b, and miRNA-206 expressions after ALS rehabilitation have been related to the clinical response after rehabilitation treatment [73,74]. Eight miRNAs are significantly dysregulated in leukocytes of ALS patients [70]. Remarkably, miRNA-338-3p has been found upregulated in leukocytes [56] and was previously shown to target the glutamate transporter EAAT2, which is responsible for the glutamate clearance described in ALS patients [75].

Circulating blood miRNAs are interesting biomarkers, because they are sufficiently stable and are able to withstand the attack of RNases, to different temperatures and pH states, and many freeze-thaw cycles [76,77]. Deregulation in circulating serum miRNAs in ALS may be a consequence of muscle denervation and degeneration, and so testing these levels during a patient’s disease progression could be useful to monitor in therapy. In the serum of ALS patients, miRNA-206, miRNA-143-3p, and miRNA-374b-5p were found to be differentially expressed compared with healthy controls. In particular, miRNA-206 and miRNA-143-3p, whereas miRNA-374b-5p was downregulated. In addition, miRNA-143-3p was upregulated while miRNA-374b-5 was significantly downregulated in the serum of sALS patients with disease progression. Studies conducted on mouse myoblast cell line (C2C12) showed that upregulation of miRNA-143-3p is negatively linked with myoblast cell differentiation and maturation, suggesting that the inhibition of miRNA-143-3p may have helpful effects on muscle wasting diseases [57]. The increased expression of miRNA-143-3p in the serum of sALS patients may be related to denervation of muscle in disease progression. Moreover, an additional analysis verified that the upregulation of miRNA-374b reduced C2C12 cell differentiation, while the inhibition of miRNA-374b expression promotes the above-mentioned event [78]. MiRNA-374b-5p is reduced in patient’s serum, most likely to compensate for the degeneration of muscles in ALS, trying to restore a balance and support muscle regeneration by promoting myoblast differentiation. Regarding deregulation in the cerebrospinal fluid of ALS patients, a study aimed to identify a set of microRNAs biomarkers. MiRNA-181a-5p, miRNA-15b-5p, and miRNA-21-5p showed the highest sensitivity and specificity in cerebrospinal fluid [40]. Particularly, in this study, miRNA-181a-5p was upregulated while miRNA-21-5p and miRNA-15b-5p were downregulated and all were related to apoptotic mechanism inhibition and cell proliferation [79]. This event could involve microglia and may contribute to neuronal damage [40].

## 4. MicroRNAs and the Treatment of ALS

Riluzole and Edaravone drugs, approved by Food and Drug Administration for ALS treatment, can only increase the survival rates of ALS patients [80]. With the innovation in genetics and pharmacology, new ALS therapies might be introduced in the clinic [81,82]. In this section, miRNA inhibition and replacement therapies for ALS are summarized, as well as approaches for miRNA delivery.

### 4.1. MiRNA Inhibition Therapy

MiRNA inhibition therapy is based on the removal of suppression operated by a miRNA on a target mRNA. The process results in the consequent increase in the expression level of the corresponding mRNA due to blocked interaction between miRNA-induced silencing complex (miRISC) and its target mRNA. The MiRNAs inhibitors most widely used are: single-stranded antisense oligonucleotides, anti-miRNA oligonucleotides (AMOs), locked nucleic acid (LNA) anti-miRs, antagomiRs, miRNA sponges, and small molecule inhibitors of miRNAs [83]. AMOs are single-stranded 2′-O-methyl-modified oligoribonucleotide fragments antisense to their target miRNAs. The Multiple-Target AMO approach is an improved AMO strategy, with AMOs composed of many antisense units in a single fragment that can silence multiple miRNAs at the same time [84]. LNA anti-miRs are nucleic-acids specifically modified to obtain high binding affinity and specificity with complementary RNA or DNA oligonucleotides, useful for both in vitro and in vivo therapies [85]. Antagomirs are a group of chemically engineered oligonucleotides that block the binding of a miRNA with the mRNA molecule [86]. Finally, small molecule inhibitors of miRNAs represent a sponge technology that consists in the synthesis of RNAs with multiple binding sites for a specific seed sequence of interest of a miRNA [87].

### 4.2. MiRNA Replacement Therapy

Supplementing specific miRNAs that are downregulated in ALS patients is another therapeutic approach that is available. The use of synthetic double-stranded miRNA mimics, pre-miRNA, or plasmid-encoded miRNAs can compensate for miRNAs loss. The miRNAs mimic approach is a method that involves the genesis of synthetic double-stranded miRNA-like RNA fragments. These RNA fragments are generated to have their 5′-ends consisting of a partly complementary motif to the selected sequence in the 3′UTR of a target. Once inside the cells, these RNA fragments mimic endogenous miRNAs and attack targets to allow the post transcription repression [88]. Agomir is a synthetic double-stranded miRNA with multiple chemical modifications that enhance its stability. Agomirs can be used to upregulate their corresponding miRNAs and to investigate the biological role of miRNA in vivo. MiRNA precursors are chemically modified single-stranded RNA fragments that are synthesized to imitate mature miRNAs. These precursors are transfected into cells using commercial reagents or electroporation. Once inside cells, miRNA precursors are cleaved by Dicer and transformed into mature miRNAs. However, as previously mentioned, miRNAs are unstable in the body due to ribonucleases and reticuloendothelial system activity. In addition, their negative charges slow the crossing of the cell membrane or vascular endothelium. Inside the cell, they are subject to endolysosomal degradation. To protect miRNAs, different delivery approaches have been formulated, as discussed in the next section.

## 5. Approaches for MiRNA Therapeutic Delivery

### 5.1. MiRNA Delivery Systems

**Figure 4:**
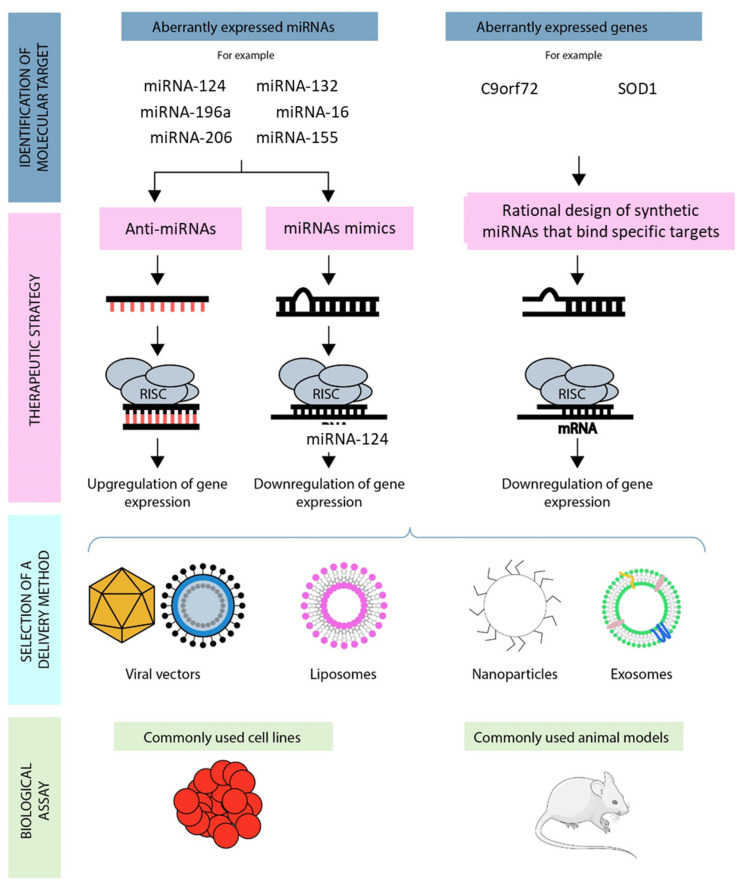
The steps used for designing and testing a miRNA-based therapeutic approach for neurodegenerative diseases. Abnormally expressed miRNAs are detected in patients’ tissues. Synthetic miRNAs are designed to target genes of interest. Specific delivery systems are designed to enter cells. The administration of miRNAs is tested in biologic models.

The scheme for designing, developing, and evaluating miRNA-based therapeutics for the treatment of ALS is depicted in Figure 4. When the miRNA candidate and its target genes are identified, the biological pathways related to the disorder must be analyzed: cell model systems and animal models should be used to verify the results of the miRNA in the development of the disorder [15].

Since miRNAs should be protected from degradation inside the bloodstream and delivered in the cells without causing any immunogenic response [89,90], oligonucleotide editing and systemic miRNAs delivery strategies were investigated. The chemical modification of miRNA oligonucleotides increases miRNA stability and averts their degradation by nucleases in the bloodstream. For instance, adding a 2′-O-methyl (2′-O Me), 2′-O-methoxyethyl or 2′-fluoro to the 2′-OH in ribosomes, intensifies the stability and the binding capacity of an anti-miRNA, and produces long-lived results in different organs [91]. As previously said, LNAs are a specific type of chemically modified RNA nucleotide analogs characterized by an elevated nuclease resistance. The systemic administration of the LNA-anti-miRNA-122 showed a dose-dependent decrease of the liver-miRNA-122 levels in mice for several weeks [92,93]. Anyway, these modified miRNAs have a small half-life due to the fast renal and hepatic clearance [94].

Indeed, one of the most important aspects related to gene-based therapy is the delivery of the therapeutic agent into the target of interest. A therapeutical agent needs to cross the biological barriers without going through degradation in the bloodstream or renal excretion [95], for this reason, specific viral or non-viral vectors have been created [96] (Figure 4).

Retrovirus, adeno-associated viruses, and lentivirus are the most used viral vectors to vehiculate genetic material. The principal advantages involve prolongation of substitution or suppression of miRNA [97] and elevated efficiency in transfection [98]. However, the success of the process can be afflicted by immune responses [99]. In retroviral vectors, the mechanism of DNA inclusion in the host chromosomes is related to the reverse-transcription of viral RNA in the cytoplasm. Retroviruses can be employed in dividing cells while lentiviruses are broadly used, considering they can infect both dividing and non-dividing cells [98]. Adeno-associated viruses infect cells, but they can replicate only if there is a helper virus [100], and they can transfer smaller genetic sequences compared to those that can bring lentiviruses (4.8 KB and 8 KB, respectively). For this reason, they are valid vectors for miRNAs transfection. Martier et al. demonstrated that miRNAs in adeno-associated virus vector serotype 5 can be used to silence C9orf72 in HEK293T cells and induce pluripotent stem cell-derived neurons [101]. Silencing of the SOD1 gene in motor neurons in vivo has also been achieved by means of adeno-associated virus vector [102].

Nevertheless, adeno-associated viruses can destruct tissues and activate inflammatory responses due to toxins production [98].

Therefore, even though viral systems have a high transfection efficiency, non-viral strategies have less toxic side effects with no restrictions linked to nucleic acid size [98]. Many kinds of materials have been exploited for the design of non-viral delivery systems.

Non-viral carriers are mainly polymeric vectors (polyetherimide, polylactic-co-glycolic acid, chitosans, and dendrimers) [103], lipid-based vectors, extracellular vesicles, and inorganic materials [104,105].

Among non-viral carriers, nanoparticles are the most widespread option for delivering exogen nucleic acids [103,106]. Nanoparticle-mediated miRNA delivery must control obstacles of the human body, such as degradation, fast renal clearance, endosomal escape pathway, and pharmacokinetics to obtain productive release [103,107,108]. The encapsulation of both miRNA mimics and anti-miRNA in nanoparticles has several advantages for the delivery in vivo, including protection of miRNA, increase of target specificity, and tissue distribution; nevertheless, encapsulation ability could decrease if nanoparticles are highly hydrophilic [109].

Liposomes are broadly used as nonviral strategies that can be used for the transfection of miRNA mimics or anti-miRNAs in vitro approaches [109]. Liposomal components can be analog to the plasmatic membrane [110] and can encapsulate nucleic acids. Liposome charge defines three classes of delivery vectors: neutral, anionic, or cationic. The cationic ones are made of positively charged lipids as well as helper lipids, which enlarge their stability and reduce toxicity [111]. Thanks to their characteristics, which include favorable interactions with negatively charged DNA, a high affinity with the plasmatic membrane, and facility of synthesis, the cationic liposomes are largely used, although their small lifespan represents the main disadvantage [98]. With the pegylation, which is the covalent linking of polyethylene glycol chains, it is possible to generate more solids complexes, as well as obtain several improvements [112]. The improved encapsulation ability and stability make lipid nanoparticles a better system compared to liposomes [113,114].

Extracellular vesicles (EVs) include exosomes and microvesicles and are natural vesicles that can deliver microRNAs and other agents to the target cell [115,116]. The loading of miRNA in extracellular vesicles can be accomplished by (i) producing a cell line that overexpresses the therapeutic miRNA or (ii) isolating EVs from patients or cell lines and using chemical or physical methods to let the miRNA enter inside [117]. The idea of using EVs is extremely attractive due to the possibility of reducing side effects such as an immune response to the carrier. However, the EVs isolation and quantification need deep technical experience [118].

Inorganic materials used for the delivery of miRNAs are mainly gold and silica nanoparticles, both stable and easily functionalizable [119]. However, these nanoparticles have been applied mostly in cancer studies and for theranostics applications. Indeed, to the best of our knowledge, miRNAs delivery by gold or silica nanoparticles in ALS has never been attempted so far.

### 5.2. Graphene-Mediated miRNA Delivery

Among many kinds of materials for drug delivery, the two-dimensional graphene [120,121,122] and its chemical derivatives [123], offer a new span of alternatives for non-viral gene delivery. The exceptional properties of graphene-based materials (GBMs) are represented by the large surface area for nucleic acids loading and limited toxicity. Moreover, carbon graphene nanoparticles, when their size is reduced, are capable of crossing biological barriers such as the blood brain barrier, which is crucial for drug delivery in the central nervous system [124,125].

**Figure 5:**
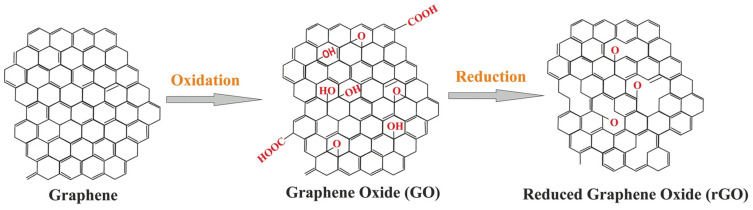
Chemical structure of graphene, GO and rGO. Reproduced with permission from [127] under Creative Commons license (http://creativecommons.org/licenses/by/4.0/, accessed on 15 December 2021).

Graphene is composed of a unique atomic layer of sp2 hybridized carbon atoms arranged in a honeycomb lattice. This atomic arrangement is responsible for most of the properties of graphene, including extreme tolerance to high temperatures [126], mechanical strength and optical and electronic properties [121]. The closest versions of graphene in the carbon-based materials family are graphene oxide (GO) and reduced graphene oxide (rGO), shown in Figure 5 [126].

GO is a chemically modified graphene with oxygen functional groups, while rGO is obtained by removing oxidized functional groups of GO by reduction. GO has easy aqueous dispersibility and biocompatibility, which has prompted studies on gene delivery, and diagnostics [128,129,130,131].

The ability of GBMs to trap nucleic acids has been broadly shown during the design of graphene-based DNA biosensors [132]. GO can load both single-stranded DNA and RNA because of hydrophobic and π–π stacking interactions between the ring structures present in nucleic acid (NA) nucleobases and the carbon lattice [133,134]. On the other hand, the surface adsorption of double-stranded NA onto GO flakes is thought to be more difficult because of its hydrophilic outer structure and the smaller accessibility of NA bases confined within the double helix structure [135]. Further operating forces such as hydrogen bonding and Van der Waals forces have been suggested to favor the association between the interface of double-stranded DNA and GO carbon rings [136,137]. It has also been proposed that fragmentary contortion of the NA double helix could promote incorporation onto the GO surface [136]. Moreover, environmental conditions, for example high salt concentrations and low pH, have been shown to considerably increase the binding capacity of double-stranded NA onto GO [138].

The GO flakes can also be exploited to separate RNA species, trapping small RNAs from complex solutions of RNA and easily separating the single-stranded nucleic acids (ssNAs) from the GO surface [139]. Different studies have demonstrated the potential of GBMs to defend NAs from enzymatic digestion. Some experiments carried out in the presence of DNAse I underlined total digestion of single-stranded DNA after 60 min of incubation. On the other side, no degradation was described in the presence of GO [140]. However, these results are subject to debate for double-stranded NA. In particular, Lei et al. showed that this protective activity was broadly dependent on salt concentrations when there was double-stranded NA [138]. rGO ability to interact with NAs has been used for ALS genosensing using DNA-hybridization and a label-free approach [141].

GBMs can be combined with other polymers to obtain multifunctional nanoparticles for gene delivery. Indeed, GBM participates in strong covalent bonding throughout carbon rehybridization from sp2 to sp3 hybrid orbital state [142]. Epoxides, carbonyls, and hydroxyls on GO give additional opportunities for amidation through epoxy ring-opening and esterification [143]. GBMs can work as electron-donating ligands in π–π bonding, but they are also able to act as electron acceptors in the case of physisorption. This mainly happens via electrostatic interactions, Van der Waals forces, and hydrogen bonds [144]. This wide range of chemical functionalization of GBMs may allow to customize pharmacokinetic properties and increase biocompatibility [145], introduce cationic agents to improve NAs loading efficiency, absorb insoluble drugs, or molecular agents that can be subject to drug resistance mechanisms [146]. Surprisingly, a photothermal effect due to the capacity of graphene to absorb near-infrared light has demonstrated an increase in the transfection efficiency. This occurs thanks to the induced heating, which locally discompose the arrangement of the lipid bilayer of cell membranes, hence making it more permeable and easing endosomal escape [147]. Lu et al. used nanoscale GO for the development of intracellular molecular probes [140].

The ability of GBMs as delivery carriers can be increased by many strategies that ameliorate the loading and delivery of the NA or increase carrier stability in the solution. The implant of cationic polymers such as polyethyleneimine (PEI) on the exterior surface of GBMs can improve gene transfection efficiency because of the positive charges that can favor the electrostatic interactions both with the NAs and the plasmatic membrane. Moreover, the positive charges can allow the release of the cargo from the endosome following the ‘proton sponge’ effect. The covalent engraftment of PEI via EDC/NHS chemistry onto both GO and rGO flakes has been studied [147,148], as well as the non-covalent electrostatic anchoring of PEI onto graphene nanoribbons [149] GO and rGO/Au composites [146]. PEI has been utilized as a non-viral gene delivery vector, nevertheless, it was compromised by its cytotoxicity, especially at high molecular weight (25 kDa) and high nitrogen-to-phosphate ratios [150]. Fusion with GBMs permits the use of low molecular weight PEI, thus decreasing its cytotoxicity [151].

Dong and colleagues have developed a strategy to recognize miRNAs into single cells using a molecular beacon with a high affinity for miRNAs complexed to PEI-graphene nanoribbons [136]. The same researchers designed an advanced graphene quantum dot-based strategy that allowed the intracellular imaging of miRNA-21 [152]. Another application of GBMs has been the silencing or downregulation of genes abnormally overexpressed by small interfering RNA (siRNA) and miRNAs delivery. Tripathi et al. designed a polyethylenimine-graphene oxide construct to vehicle a green fluorescent protein (GFP)-encoding plasmid-DNA and were able to silence its expression thanks to the delivery of an anti-GFP siRNA [153]. Gene silencing by an analogous GO–PEI:siRNA complex has been demonstrated by Huang et al. [154] to downregulate its intracellular target C-X-C Motif Chemokine Receptor 4, a chemokine receptor broadly related to cancer metastasis. This effect decreases the migratory ability of cancer cells. Dong and colleagues studied the opportunity of delivering two antisense probes against different targets (miRNA-21 and survivin) in the same vector, which arises in a synergistic effect against the growth of HeLa cancer cells [152]. In the case of induction of gene expression, the photothermal abilities of GBMs can also increase siRNA delivery. Feng et al. improved the intracellular internalization of a siRNA against the protooncogene Polo-like kinase 1, which results in downregulating the mRNA target and the protein level [147].

The interaction of GBMs with neurons and astrocytes is still poorly investigated and unclear, due to the variable methods for GBM production that influence the oxygen content, lateral size, contaminants, and number of layers [155]. In one of the earlier studies, Defterali et al. using rGO showed good neuronal and glial biocompatibility [156,157]. It seems plausible that carbon nanomaterial might modulate neuroinflammation, a common feature of neurodegenerative diseases [158]. Alternatively, graphene or GBM, in general, can be functionalized with other inorganic materials or biopolymers to ensure biocompatibility and stability. As an example, GO functionalized with silica nanoparticles induces human nerve stem cell differentiation and axonal alignment [159]. GO coated with cationic lipids has been used to improve cells transfection with DNA, and the same strategy could be used for miRNA delivery [160]. Interestingly, even if GBM nanoparticles are still not investigated in ALS therapy, successful delivery of pharmaceutics has been obtained for other neurodegenerative diseases such as Parkinsons’ and focal brain injury, paving the way for further research in the field [161,162].

## 6. Conclusions

ALS muscle atrophy, weakness, fasciculations, and spasticity impair patients’ quality of life and lead to death. Compared to other neurodegenerative diseases, such as Alzheimer’s and Parkinson’s disease, the dysfunction of specific miRNAs involved in the pathogenesis of ALS is less easy to detail. However, emerging studies have confirmed the role of miRNAs in the pathogenesis of ALS in patients. Thus, it could be useful to correct the abnormal expression of miRNAs related to ALS.

With this aim, many therapeutics approaches can be designed. The generic protocol for designing and testing a miRNA-based therapeutic approach to treat ALS consists first of the detection of abnormally expressed miRNAs. After that, these ncRNAs have to be regulated by inhibiting or enhancing their action. With this aim, specific delivery systems can be designed using viral or non-viral systems. The viral systems are characterized by a higher transfection efficiency but can be highly toxic and immunogenic. The nonviral carriers have lower delivery efficiency but are safer. Among these, the recently discovered two-dimensional, mono-atomic carbon material graphene and its chemical derivatives, could be exploited to reduce neuroinflammation and cross the biological barriers of the central nervous system while loading nucleic acids. Nevertheless, the application of graphene in ALS is still lacking. This review aims at highlighting the opportunity offered by graphene in the field to promote future ALS research focused on this bidimensional material.

## Figures and Tables

**Table 1:** Aberrant miRNAs in ALS.

MiRNA		Model	Function and Changes	Ref
Animal model	miRNA-125b 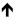	SOD1-G93A mouse	Increase of TNFα transcription by interfering with the STAT3 pathway	[44]
	miRNA-124 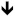		Aberrant modulation of astrocytic differentiation by targeting Sox2 and Sox9	[45]
	miRNA-128 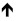		Aberrant modulation of the excitatory amino acid transporter 2	[46]
	miRNA-193b-3p 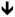		Stimulation of cell death by targeting tuberous sclerosis 1 (TSC1)	[47]
	miRNA-375-3p 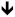	Wobbler mouse	Inefficient inhibition of p53, increasing ROS production, and induction of cell death	[48]
ALS patients	miRNA-146* 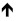	sALS	Interaction with NFL mRNA, and suppression of its expression	[49]
	miRNA-524-5p 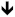 miRNA-582-3p 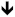	sALS	Aberrant regulation of NFL mRNA 3′UTR	[49]
	miRNA-375 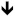	FUS mutant ALS	Aberrant targeting of the apoptotic factor ELAV-like protein 4	[50]
	miRNA-494-3p 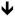	C9orf72 mutant ALS	Aberrant regulation of semaphorin 3A	[51]
	miRNA-223-3p 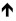 miRNA-338-39 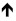 miRNA-326 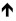	sALS	Altered homeostasis in the neuromuscular junction	[52]
	miRNA-1 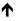 miRNA-19a-3p 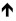 miRNA-133a-3p 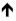 miRNA-133b 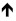 miRNA-144-5p 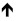 miRNA-192-3p 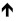 miRNA-192-5p 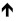	fALS and sALS	Altered homeostasis in the serum	[53]
	Let-7d3p 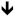 miRNA-139-5p 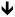 miRNA-320a 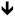 miRNA-320c 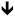 miRNA-320b 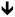 miRNA-425-5p 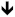	sALS and fALS	Altered homeostasis in the serum	[53]
	miRNA-1825 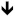	sALS and fALS	Overtranslation of tubulin-folding cofactor b, which contributes to the degradation of tubulin alpha-4A	[54]
	miRNA-424 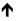 miRNA-206 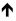	ALS	Altered homeostasis in the plasma and in the skeletal muscle	[55]
	miRNA-338-3p 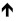	sALS	Altered homeostasis in leukocytes	[56]
	miRNA-206 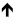	sALS	Altered homeostasis in the serum (no significant change in the serum over time)	[57]
	miRNA-143-3p 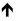 miRNA-374b-5p 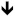	sALS	Altered homeostasis in the serum (significant change in the serum over time)	[57]
	miRNA-181a-5p 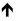 miRNA-21-5p 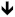 miRNA-15b-5p 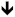	ALS	Alteration of apoptotic mechanism inhibition and cell proliferation	[40]

## Data Availability

Not applicable.
